# New insights into CAR T cell-mediated killing of tumor cells

**DOI:** 10.3389/fimmu.2022.1016208

**Published:** 2022-09-15

**Authors:** David Espie, Emmanuel Donnadieu

**Affiliations:** ^1^ Université Paris Cité, CNRS, INSERM, Equipe Labellisée Ligue Contre le Cancer, Institut Cochin, Paris, France; ^2^ CAR-T Preclinical Development Department, Invectys, Paris, France

**Keywords:** CAR (chimeric antigen receptor) T cells, tumor cell, interferon gama (IFNγ), adhesion, immune synapse, cytotoxicity

## Abstract

Adoptive transfer of T cells genetically engineered to express chimeric antigen receptors (CAR) has demonstrated striking efficacy for the treatment of several hematological malignancies, including B-cell lymphoma, leukemia, and multiple myeloma. However, CAR T-cell efficacy has been very limited in most solid tumors. In this context, it is of paramount importance to understand the determinants that condition CAR T-cell success versus failure. To control tumor growth, CAR T cells need to form conjugates with their targets *via* the assembly of an immunological synapse. Here, we review recent advances showing that the adhesion between CAR T cells and cancer cells from solid tumors strengthens over time in an IFNγ- and ICAM-1-dependent manner, resulting in CAR T cell-mediated killing. We discuss how these findings can be exploited to increase the efficacy of the CAR T-cell strategy against solid tumors.

## Introduction

### CAR T cell therapy fails in solid tumors

CAR T-cell therapy has shown considerable promise for hematologic malignancies. To date, six CAR T products have been approved by the Food and Drug Administration (FDA) in the United States, targeting leukemia, lymphoma, and multiple myeloma (for a review see (1)). Despite this success, the field is facing many challenges such as antigen heterogeneity and toxicity issues (2). Moreover, solid tumors are, with some exceptions (3), resistant to CAR T cells. Understanding mechanisms of resistance to CAR T cell therapy in solid tumors is therefore a key challenge and opportunity.

### Known resistant mechanisms to CAR T cells

Over the last years, several determinants have been considered important in controlling CAR T cell efficacy (4). Those that retain the most attention include the expression of the target antigen, the affinity of the CAR as well as the nature of the costimulatory domain. Loss of the target antigen is nearly always associated with patient relapses (5) and many efforts are being made to increase antigen sensitivity (6, 7). However, additional factors can also lead to resistance. CAR T-cell intrinsic properties controlled by metabolism and differentiation parameters are key in the ability of infused T cells to proliferate and persist or not within the host. In addition, recent articles have shown that tumor cells can resist CAR T-cell killing in various ways such as the capacity to repair their membranes following a ‘hit’ from cytotoxic T-cells (8) as well as the presence of mutations in apoptosis pathways (9). Lastly, the immunosuppressive tumor microenvironment (TME) may also generate resistance to CAR T-cell treatments, especially in solid tumors (10).

### Role of adhesion/costimulatory molecules in T cell-tumor cell interaction

To control tumor growth, CAR T cells need to form productive conjugates with their targets *via* the assembly of an immunological synapse (11). This complex cell-cell interaction structure orchestrates T cell activation and triggers the polarized release of cytotoxic granules, enriched with perforin and granzyme B, which eventually induce target cell death. Up to now, little is known about the mechanisms that regulate CAR T-cell interaction with tumor cells. Previous studies performed with non-modified T cells have underlined that, in addition to the recognition of specific peptide-major histocompatibility complex molecules *via* the T-cell receptor (TCR), engagement of adhesion and costimulatory molecules with their respective ligands is mandatory to trigger efficacious antitumor T-cell activities. CD2, which binds to CD58 (LFA-3) on target cells acts as an adhesion/costimulatory molecule that provides signals to amplify TCR signaling (12). Integrins, in particular lymphocyte function-associated antigen-1 (LFA-1, CD11a/CD18 or αLβ2) and CD103 (αEβ7), also play important roles in T cell-target cell adhesion and signaling through interaction with their respective ligands, intercellular adhesion molecule-1 (ICAM-1 or CD54) and the epithelial cell marker E-cadherin (13, 14). In native T cells, the adhesive properties of integrins are regulated *via* conformational activation and clustering, initiated by an “inside-out” signaling process emanating at least in part from the TCR (15).

In a cancer context, previous studies have reported downregulation of adhesion molecules on tumor cells and tumor-infiltrating T cells (TILs) contributing to the defective formation of a productive synapse (12, 16, 17). Non-lytic TILs purified from the murine adenocarcinoma MC38 had reduced cell surface expression of adhesion molecules CD2, CD8, and LFA-1 (17). Likewise, in patients with colorectal, endometrial or ovarian cancer, CD8+ TILs displayed low expression of CD2. Finally, the adhesion molecule ICAM-1 is frequently downregulated by cancer cells which might prevent CD8 T cells to kill their targets (16).

## Importance of the LFA-1 - ICAM-1 axis in CAR T-cell responsiveness

Within the CAR-T immune synapse, the relevance of adhesion/costimulatory molecules has received little attention so far. However, very recent publications are putting a new light on determining the role of adhesion molecules and in particular on ICAM-1.

We have found that CAR T-cell initial activation was strongly dependent on the expression level of ICAM-1 on tumor cells (18). By comparing the ability of CD20 and EGFR CAR T cells to increase intracellular Ca^2+^ (Ca^2+^)_i_ during interaction with their respective targets - B lymphoma and tumor pancreatic cell lines - we reported that EGFR CAR T cells presented fewer responses than CD20 CAR T cells. Using an antibody screen to identify the origin of this differential CAR T cell responsiveness, ICAM-1 was found to be highly expressed at the surface of the Raji B lymphoma cell line whereas in two different carcinoma cell lines (BxCP3 and EGI-1) the surface expression of ICAM-1 was very low. In addition, analyzing malignant B cells from chronic lymphocytic leukemia patients our data indicated that the percentage of activated CD20 CAR T cells was positively correlated with the amount of ICAM-1 on cancer cells.

The role of LFA-1/ICAM-1 interaction in CAR T-cell activation was also highlighted in two recent studies. In the first, the authors exploited the property of the extracellular magnesium (Mg^2+^) to bind to LFA-1 and to stabilize its active conformation. Under low Mg^2+^ levels, CAR T-cell activation and cytotoxicity against tumor cells were considerably reduced. Most importantly, in lymphoma patients treated with CD19 CAR T cells, low serum Mg^2+^ levels correlated with a worse prognosis (19). Secondly, the importance of ICAM-1 expression on cancer cells for CAR T-cell activation was also revealed in a CRISPR-based screen performed in a multiple myeloma cell line. Knock out of ICAM-1 gene in tumor cells led to resistance to BCMA CAR T cells (20).

In line with these findings, it was shown that blocking LFA-1/ICAM-1 interaction with gene targeting strategies decreased CAR T-cell interaction with tumor cells as well as cytotoxicity and tumor growth control in mouse models (18, 21).

## IFNγ produced by antigen-stimulated CAR T cells upregulates ICAM-1 and facilitates productive interaction with tumor cells

**Figure 1:**
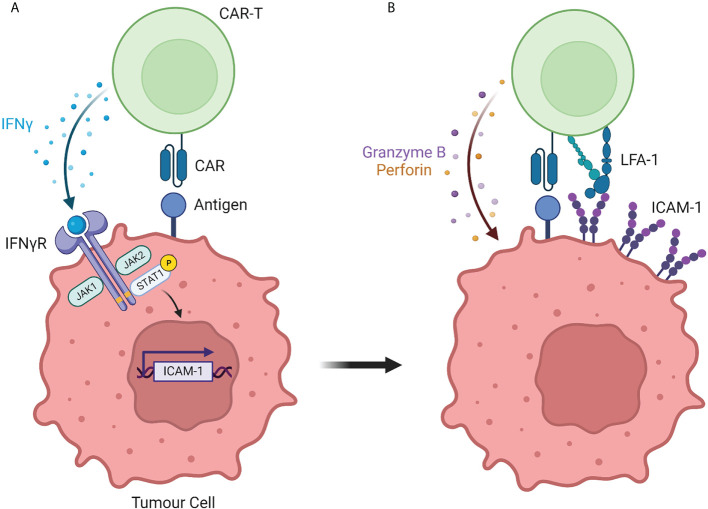
The interaction between CAR T cells and solid tumor cells is controlled by the IFNγ/ICAM-1 axis. **(A)** During initial interaction with solid tumor cells, CAR T cells secrete IFNγ which induces the transcription of the adhesion molecule ICAM-1. **(B)** ICAM-1 expression by solid tumor cells strengthens the adhesion with CAR T cells, resulting in tumor cell killing. Created with BioRender (Biorender.com)

The importance of the LFA-1/ICAM-1 axis in CAR T-cell responsiveness was somewhat expected in light of its role in controlling the interaction between non-modified T cells and their targets. More surprising was the ability of CAR T cells to progressively strengthen their interaction with tumor cells that initially expressed low levels of ICAM-1 and to do so in an IFNγ-dependent manner (**Figure 1**). Such finding was made independently by several labs using different strategies.

We suspected the importance of IFNγ by performing a kinetic experiment during which fresh tumor slices were exposed for different times to EGFR CAR T cells. After 30 minutes and in agreement with our previous findings (22), engineered T cells were found in the stroma or in contact with cancer cells localized at the periphery of tumor islets. Strikingly, 20 hours later CAR T cells redistributed to tumor islets (18). This T cell enrichment in tumor cell regions was associated with a marked increase in ICAM-1 expression on tumor cells and was inhibited by a blocking monoclonal IFNγ antibody. Based on these results we proposed a two-step process of CAR T cell interaction with tumor cells. First, CAR T cells get activated at the periphery of tumor islets and start producing IFNγ. This inflammatory cytokine, in line with prior studies (23) upregulates ICAM-1 on tumor cells facilitating the formation of productive conjugates between CAR T cells and their targets.

By conducting a genome-wide CRISPR knockout (KO) screen in human glioblastoma cells, Larson et al. demonstrated the importance of IFNγ-signaling in tumor cells in CAR T-cell-mediated killing (24). After two days of co-culture with EGFR CAR T cells, resistant clones of tumor cells were enriched for loss of genes involved in IFNγ-mediated signaling, such as IFNGR1 and JAK1. The importance of the IFNγ pathway was confirmed by generating IFNγR1 KO glioblastoma cells which were more resistant to CAR T cell-mediated killing than wild-type tumor cells. The Maus lab generalized these findings to other solid tumors by performing *in vitro* but also *in vivo* experiments in xenografted mice. Using transcriptional profiling, Larson et al. found that tumor cells lacking IFNγR1 had lower upregulation of ICAM-1 after exposure to CAR T cells. Additional experiments showed that IFNγR signaling on tumor cells was required for sufficient adhesion of CAR T cells to mediate productive cytotoxicity. Here again, the connection between IFNγ produced by antigen-primed CAR T cells and the subsequent productive interaction leading to tumor cell death was demonstrated.

Strikingly, this mechanism turned out to be true for many solid tumors but not for hematological malignancies (24, 25). Conversely, IFNγ signaling in B-cell lymphoma has been associated with CAR T cell failure (26).

## Exploiting IFNγ - adhesion pathways to make more efficient CAR T cells against solid tumors

For this process to be optimal, a large number of engineered T cells should infiltrate tumor islets to produce a high amount of IFNγ acting on cancer cells. In solid tumors, these mechanisms can be altered in several ways. One can mention, the presence of obstacles in the tumor stroma that impede T cells from reaching cancer cells (27) and will limit the amount of IFNγ produced.

Here, we review strategies that can be developed targeting the IFNγ and adhesion pathways.

The control of IFNγ production can be harnessed in different ways. First, the presence of a sufficient number of CAR T cells derived from the CD4 T cell subset is important knowing the propensity of helper T cells to produce IFNγ. Accordingly, results from previous clinical trials have shown that the therapeutic efficacy was optimal with defined infused CD4/CD8 cell ratios and superior to that of the subset alone (28). Along the same lines, T cells expressing CARs with a CD28 costimulatory domain have been shown to release higher quantities of IFNγ than T cells expressing 4-1BB-costimulated CARs (29). A comparison of the effects of CD28 and 4-1BB costimulatory domains in CAR T- cell activation and interaction with tumor cells remains to be conducted.

Adhesion molecules represent other promising targets to boost productive interactions between CAR T cells and tumor cells. The enhancement of CAR T-cell cytotoxic activity against tumor cells through the activation of LFA-1 with external Mg^2+^ constitutes an important proof-of-concept for such a strategy (19). However, this approach possesses limited clinical translation as it is difficult to fine-tune external Mg^2+^ levels in tumors. A small molecule activator (7HP349) of the integrins LFA-1 and VLA-4 attracts attention. This compound has recently been shown to promote T cell recruitment in cold tumors and to increase the efficacy of CTLA-4 blockade in mice (30). In addition, several negative regulators of LFA-1 activity have been identified (31) offering the possibility of genetically targeting (CRISPR/CAS9) these determinants in CAR T cells to boost their adhesion with tumor cells.

## Questions and conclusion

Several questions remain regarding the unique characteristics of this mechanism.

### What are the roles of other IFNγ-induced genes in controlling CAR T cell efficacy?

IFNγ receptor signaling leads to an increase in the production of CXCR3 ligands, namely CXCL9, 10 and 11 which have the potential to promote the avidity of LFA-1 on T cells as well as to attract other immune effector cells to the tumor site. In this context, Using an ex vivo 3D tumor model Ronteix et al. (32) have shown that the first T cells contacting tumor cells initiated a positive feedback loop that accelerated the recruitment of other T cells to the tumor spheroid in agreement with the secretion of a T cell-attractant factor.

Apart from acting on tumor cells, IFNγ produced by antigen-stimulated CAR T cells has been shown to reprogram the tumor microenvironment leading to beneficial effects on CAR T cells (33). Of note, IFNγ can spread long distances (several hundred microns) acting on tumor cells not expressing the antigen (34, 35) with the potential to promote antigen spread and the generation of tumor-specific T-cell responses.

On the other hand PD-L1 upregulation by IFNγ can contribute to immune evasion and approaches to combining CAR T cells with PD-1/PD-L1 blockade have produced promising results even in hard-to-treat cancers (36).

### What is the structure of the synapse formed between CAR T cells and tumor cells?

Initial studies have reported a disorganized immunological synapse formed between CAR T cells and tumor cells (37) which might reflect the initial, suboptimal conjugates between both cell types. Moreover, whereas CAR’s simple architecture affords much flexibility in clinical applications it limits the extent to which CAR reproduce the complexities of the TCR signaling responses (38, 39).

Based on recent findings discussed here showing a strengthening of the adhesion, we assumed that the synapse formed between CAR T cells and tumor cells will evolve over time and get structured due to ICAM-1 upregulation and interaction with LFA-1. Of interest, integrins including LFA-1 have been shown to exert forces at the synapse enabling correct degranulation of cytotoxic T cells (40).

### What is the clinical relevance of these findings?

All studies discussed here were performed in preclinical models. Due to the presence of molecular aberrations in the IFNγ signaling pathways, many cancer patients do not respond to immune checkpoint blockade strategies (41). Whether such mutations contribute to explaining CD19 CAR T-cell failure in patients remain to be demonstrated.

### Are other adhesion molecules important in controlling CAR T cell-mediated killing?

Although the aforementioned studies focused on the role of ICAM-1, other adhesion pathways might also be operative in CAR T cell-target cell interaction. One group has recently reported that the loss of the adhesion/costimulatory molecule CD58 (the ligand of CD2) at the surface of tumor cells from large B-cell lymphoma patients is associated with CD19 CAR T-cell failure (42). Likewise, CD58 has also been identified in a recent CRISPR–Cas9 loss of function screen performed in a CD19 CAR T cell-leukemia cell co-culture model (43). Notably, CD58 is frequently downregulated not only in large B-cell lymphomas but also in multiple other lymphoid malignancies (44) suggesting that this tumor cell-intrinsic resistance mechanism might be frequent.

In carcinomas, we reported that EGFR CAR T cells preferentially get activated in contact with tumor cells localized at the periphery of tumor islets (18). Although low for ICAM-1, these tumor cells highly express the integrin α6β4. A blocking anti-β4 antibody partially decreased the initial CAR T-cell activation during interactions with peripheral tumor cells which suggests a role of this integrin (18). Clearly, more investigations are needed to confirm the participation of this adhesion molecule as well as other adhesion pathways (e.g., CD103 (αEβ7)) during the initial CAR T cell-tumor cell interaction.

In conclusion, these recent findings highlight an emerging theme that we should not just consider CAR T cells as only killers but also as modifiers of the TME through the production of IFNγ. In turn, these changes result in improved CAR T-cell antitumor activities. Overall, these recent reports demonstrate the importance of studying dynamic T cell-tumor cell interactions in identifying novel mechanisms to boost the efficacy of the CAR T-cell strategy against solid tumors.

## Author contributions

DE and ED conceived, wrote the article and designed the figure. All authors read and approved the final manuscript.

## Funding

This research was funded by Ligue National Contre le Cancer, grant number EL2020.LNCC/EmD.

## Conflict of interest

Author DE was employed by Invectys.

The remaining author declares that the research was conducted in the absence of any commercial or financial relationships that could be construed as a potential conflict of interest.

## Publisher’s note

All claims expressed in this article are solely those of the authors and do not necessarily represent those of their affiliated organizations, or those of the publisher, the editors and the reviewers. Any product that may be evaluated in this article, or claim that may be made by its manufacturer, is not guaranteed or endorsed by the publisher.

## Abbreviations

CAR, chimeric antigen receptor; IFN, interferon; TIL, tumor-infiltrating lymphocytes; ICAM, intercellular adhesion molecule; TCR, T cell receptor; TME, tumor microenvironment.
